# Enhancing 3D Face Recognition: Achieving Significant Gains via 2D-Aided Generative Augmentation

**DOI:** 10.3390/s25165049

**Published:** 2025-08-14

**Authors:** Cuican Yu, Zihui Zhang, Huibin Li, Chang Liu

**Affiliations:** 1 Department of Hepatobiliary Surgery, The Second Affiliated Hospital of Xi’an Jiaotong University, Xi’an 710004, China; cuicanyu@xjtu.edu.cn; 2 Department of Computing, Hong Kong Polytechnic University, Hong Kong 999077, China; zihui.zhang@connect.polyu.hk; 3 School of Mathematics and Statistics, Xi’an Jiaotong University, Xi’an 710049, China

**Keywords:** 3D face recognition, generative augmentation, pattern recognition

## Abstract

The development of deep learning-based 3D face recognition has been constrained by the limited availability of large-scale 3D facial datasets, which are costly and labor-intensive to acquire. To address this challenge, we propose a novel 2D-aided framework that reconstructs 3D face geometries from abundant 2D images, enabling scalable and cost-effective data augmentation for 3D face recognition. Our pipeline integrates 3D face reconstruction with normal component image encoding and fine-tunes a deep face recognition model to learn discriminative representations from synthetic 3D data. Experimental results on four public benchmarks, i.e., the BU-3DFE, FRGC v2, Bosphorus, and BU-4DFE databases, demonstrate competitive rank-1 accuracies of 99.2%, 98.4%, 99.3%, and 96.5%, respectively, despite the absence of real 3D training data. We further evaluate the impact of alternative reconstruction methods and empirically demonstrate that higher-fidelity 3D inputs improve recognition performance. While synthetic 3D face data may lack certain fine-grained geometric details, our results validate their effectiveness for practical recognition tasks under diverse expressions and demographic conditions. This work provides an efficient and scalable paradigm for 3D face recognition by leveraging widely available face images, offering new insights into data-efficient training strategies for biometric systems.

## 1. Introduction

In recent years, deep learning, particularly deep convolutional neural networks (DCNNs), has led to significant breakthroughs in computer vision, pushing the limits of tasks such as image classification, segmentation, and object detection [1,2,3]. These advances have also profoundly influenced the field of face recognition. In the 2D domain, the availability of large-scale annotated datasets has been a driving force behind the development of high-performance DCNN-based face recognition systems. For example, Google’s FaceNet [4] was trained using over 200 million face images from more than 8 million unique identities, while Parkhi et al. [5] curated the VGGFace dataset, consisting of 2.6 million face images. These massive datasets provide not only extensive coverage of intra-class variation but also the statistical diversity necessary for learning robust, generalizable face representations.

However, despite the remarkable progress in 2D face recognition, extending these deep learning approaches to 3D face recognition remains a significant challenge. The lack of large-scale, high-quality 3D face datasets is a key bottleneck. Collecting 3D face scans is time-consuming, expensive, and generally requires specialized hardware and controlled conditions. As a result, existing publicly available 3D datasets are orders of magnitude smaller than their 2D counterparts. For instance, FRGC v2 contains only 4007 3D scans from 466 subjects, while Lock3DFace [6] includes 5711 video sequences from 509 individuals, with depth data acquired using Kinect sensors that offer limited spatial and depth precision. This data scarcity poses a critical obstacle to large-scale training of 3D face recognition models, and motivates alternative strategies for scalable data augmentation.

To address the scarcity of 3D face data, existing augmentation strategies can be broadly categorized into transformation-based and synthesis-based methods. Transformation-based approaches apply operations such as rotation, scaling, masking, flipping, and noise injection to perturb existing 3D face scans. These methods are simple, widely used, and effective for introducing pose or geometric variation, but they cannot generate new facial expressions or identities. Representative works include random rigid and affine transformations on 3D scans or their depth maps [7,8]. Synthesis-based methods aim to generate novel 3D faces with new expressions or identities, typically through 3D Morphable Models (3DMMs) [7] or Gaussian Process Morphable Models (GPMMs) [9], by sampling shape and expression coefficients. While such approaches enhance identity diversity, the inherent linearity of these models often restricts the discriminative capacity of the synthesized faces. Other works explore interpolation-based identity synthesis [10], or leverage 3D-aided GAN frameworks to simulate variations [11]. However, challenges such as label noise, limited expression realism, and reliance on dense correspondence persist. Overall, despite moderate gains, most existing approaches remain confined to the 3D domain and are ultimately limited by the diversity and quantity of real 3D scans.

Recognizing the abundance and accessibility of 2D face images, a promising alternative direction is to reconstruct 3D face geometry from 2D images, enabling a 2D-to-3D augmentation pipeline. Although significant progress has been made in 3D face reconstruction from single or multiple 2D images, its potential as a scalable training strategy for deep 3D face recognition remains largely underexplored. In particular, few works have systematically investigated whether 3D faces reconstructed from 2D images can effectively support the training of high-performance 3D face recognition models. To bridge this gap, we propose a novel 2D-aided deep 3D face recognition framework that exploits large-scale 2D face datasets to train a high-performance 3D face recognition model. Our key idea is to shift the burden of data collection from the expensive 3D domain to the readily available 2D domain. Specifically, we employ a lightweight 3D face reconstruction model to convert massive collections of 2D face images into 3D face surfaces. These reconstructed 3D faces are projected into structured normal component images that preserve spatial and identity cues, and are compatible with standard DCNN architectures. The normal component images are subsequently used to fine-tune a deep face recognition model originally pre-trained on 2D face data. This design serves two purposes: (1) it leverages the discriminative capacity of large-scale 2D learning, and (2) it introduces valuable 3D spatial cues through geometry-aware representations. In contrast to traditional 3D-to-3D augmentation methods, our approach provides a scalable and cost-effective pathway to expand 3D training data using existing 2D resources. Our framework is modular and agnostic to the choice of 3D reconstruction model, enabling future improvements through more advanced reconstruction techniques.

We validate our approach on four widely used 3D face benchmarks: BU-3DFE, FRGC v2, Bosphorus, and BU-4DFE. Extensive experiments demonstrate that our method consistently achieves competitive performance across datasets. Notably, despite being trained solely on reconstructed 3D faces from 2D images, our model performs on par with or surpasses state-of-the-art methods trained on real 3D scans. Compared to a baseline model trained only on 2D images, our method improves rank-1 accuracy by 22.0% on BU-3DFE, 10.9% on FRGC v2, and 15.8% on Bosphorus. These results underscore the effectiveness of our 2D-aided training strategy and highlight its potential as a scalable solution for 3D face recognition in scenarios where high-quality 3D data are scarce or unavailable. To our knowledge, this is one of the first works to systematically validate that 3D faces reconstructed from 2D images can effectively support high-performance 3D face recognition, even without access to real 3D training data.

## 2. Proposed Method

**Figure 1:**
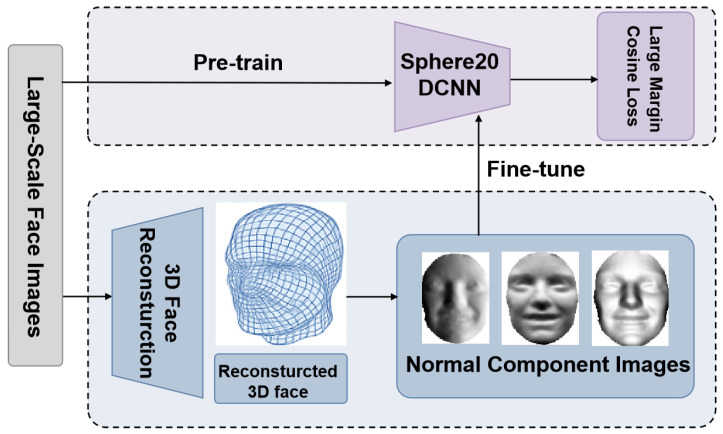
Framework of the proposed 2D-aided 3D face recognition method.

The proposed 2D-aided deep 3D face recognition framework comprises four key stages: (1) Pre-training: We first pre-train a deep face recognition model using a large number of readily available 2D face images to learn robust facial feature representations. (2) Three-Dimensional Face Reconstruction: A large-scale 3D face dataset is reconstructed from those 2D face images, and their corresponding normal component images are generated. This step ensures the transformation of the 2D data into meaningful 3D representations. (3) Fine-tuning: The pre-trained deep face recognition model is fine-tuned using the normal component images derived from the reconstructed 3D faces. (4) Identification: Three-dimensional face identification is then achieved by comparing and integrating the scores from the extracted deep features of probe and gallery three-dimensional face scans. We provide a detailed explanation of each component of our approach in the following subsections. Figure 1 demonstrates the framework of the proposed method.

### 2.1. Pre-Training the Face Recognition Model with 2D Images

To address the scarcity of large-scale 3D face datasets, we adopt a pre-training strategy based on 2D face images. We use the SphereFace [12] architecture, a 20-layer convolutional neural network (Sphere20) tailored for face recognition. The standard output layer can be written as

(1) $${\hat{y}}_{ij}=\mathrm{softmax}\left(W\cdot h\left(x_{i}\right)+b\right),$$

where $x_{i}$ is the input 2D face image, $h\left(x_{i}\right)\in R^{d}$ is the deep feature embedding extracted by the convolutional backbone, $W\in R^{C\times d}$ is the weight matrix of the final fully connected layer, with *C* denoting the number of training classes, $b\in R^{C}$ is the bias term, and the softmax function maps the output to class probabilities.

The standard cross-entropy loss function is employed during pre-training. Given a set of 2D training images with class labels, the objective is to maximize the posterior probability of the correct class:

(2) $$L=-\sum_{i=1}^{N}\sum_{j=1}^{C}y_{ij}\log\left({\hat{y}}_{ij}\right),$$

where *N* denotes the total number of training samples in a mini-batch, *C* represents the number of classes, $y_{ij}$ is the ground-truth label, ${\hat{y}}_{ij}$ is the predicted probability that the *i*-th sample belongs to class *j*.

Pre-training deep face recognition on large-scale 2D face datasets allows the model to learn general face representations, which significantly improves the convergence speed and generalization ability when fine-tuned in the 3D domain. This strategy helps mitigate the scarcity of large-scale annotated 3D facial data by transferring knowledge from abundant 2D face image data.

### 2.2. Three-Dimensional Face Reconstruction with ExpNet

To facilitate large-scale training for 3D face recognition, we reconstruct 3D facial geometries from 2D images using the ExpNet framework [13]. ExpNet employs a deep neural network to directly regress the parameters of a 3D Morphable Model (3DMM) from a single input image, thereby eliminating the need for iterative, landmark-based 3DMM fitting procedures that are computationally intensive and prone to alignment errors. ExpNet models the reconstructed 3D face as a linear combination of a mean shape, a shape basis, and an expression basis:

(3) $$S^{\prime }=\hat{S}+S\vec{\alpha }+E\vec{\eta },$$

where $\hat{S}\in R^{3n}$ is the mean 3D face shape, *n* denotes the number of vertices, $S\in R^{3n\times 99}$ is the principal component shape basis, learned from neutral 3D face scans, $E\in R^{3n\times 29}$ is the expression basis, capturing variations in facial expressions. $\vec{\alpha }\in R^{99}$ and $\vec{\eta }\in R^{29}$ are the shape and expression coefficient vectors predicted by the face reconstruction network.

The face reconstruction network is trained using a regression loss that supervises the predicted coefficients with respect to ground-truth 3DMM parameters, typically obtained via multi-view or synthetic data. By formulating the reconstruction as a direct regression task, ExpNet achieves efficient inference and generalizes well to in-the-wild 2D face images. Compared to conventional 3DMM fitting approaches, which rely on detecting facial landmarks and optimizing via iterative energy minimization, ExpNet offers significant advantages in terms of speed, scalability, and robustness to occlusion or pose variation. These properties make it well-suited for constructing large-scale 3D face datasets from existing 2D collections.

Our pipeline is modular: the reconstruction component can be substituted with other 3D face reconstruction models [3] as long as the reconstructed meshes retain sufficient structural fidelity. In this work, we adopt ExpNet for its computational efficiency and compatibility with large-scale 2D pre-training scenarios.

### 2.3. Fine-Tuning the Face Recognition Model with Normal Component Images

To effectively leverage the pre-trained deep face recognition model for 3D face recognition, we introduce a normal-based representation that bridges the modality gap between 3D geometric surfaces and 2D image-based models. In our framework, the 3D face meshes regressed by ExpNet are first converted into dense range images—structured grids of size m×n, where each cell encodes the 3D coordinate (x,y,z) of a surface point. This rasterized representation $P\in R^{m\times n\times 3}$ not only facilitates pixel-wise normal estimation via local plane fitting [14], but also aligns naturally with the input format expected by 2D convolutional networks.

Based on this representation, we compute three 2D images, referred to as normal component images (NCIs), which describe the surface orientation along the *x*-, *y*-, and *z*-directions, respectively. These NCIs serve as modality-aligned inputs for fine-tuning the pre-trained model, enabling effective 3D face recognition using 2D deep features.

Given a 3D face surface $P\in R^{m\times n\times 3}$ parameterized as a dense vertex grid,

(4) $$P={\left[p_{ij}\left(x,y,z\right)\right]}_{m\times n}={\left[p_{ijk}\right]}_{m\times n\times \{x,y,z\}},$$

where $p_{ij}\left(x,y,z\right)={\left(p_{ijx},p_{ijy},p_{ijz}\right)}^{T},\left(1\leq i\leq m,1\leq j\leq n,i,j\in Z\right)$ represents the 3D coordinates of the point $p_{ij}.$ Let its unit normal vector matrix (m×n×3) be

(5) $$N(P)={\left[n\left(p_{ij}\left(x,y,z\right)\right)\right]}_{m\times n}={\left[n_{ijk}\right]}_{m\times n\times \{x,y,z\}},$$

where $n\left(p_{ij}\left(x,y,z\right)\right)={\left(n_{ijx},n_{ijy},n_{ijz}\right)}^{T},\left(1\leq i\leq m,1\leq j\leq n,i,j\in Z\right)$ denotes the unit normal vector of $p_{ij}.$ As described in [14], the normal vector N(P) of range image P can be estimated by fitting local plane. That is, for each point $p_{ij}\in P$, its normal vector $n(p_{ij})$ can be estimated as the normal vector of the following local fitted plane:

(6) $$S_{ij}:n_{ijx}q_{ijx}+n_{ijy}q_{ijy}+n_{ijz}q_{ijz}=d,$$

where ${\left(q_{ijx},q_{ijy},q_{ijz}\right)}^{T}$ represents any point within the local neighborhood of point $p_{ij}$ and $d=n_{ijx}p_{ijx}+n_{ijy}p_{ijy}+n_{ijz}p_{ijz}.$ In this paper, a neighborhood of 5×5 window is used. To simplify, each normal component in Equation (5) can be represented by an m×n matrix:

(7) $$N(P)=\left\{\begin{array}{c} N_{x}={\left[n_{ij}^{x}\right]}_{m\times n},\\ N_{y}={\left[n_{ij}^{y}\right]}_{m\times n},\\ N_{z}={\left[n_{ij}^{z}\right]}_{m\times n}. \end{array}\right.$$

where $∥{\left(n_{ij}^{x},n_{ij}^{y},n_{ij}^{z}\right)}^{T}{∥}_{2}=1.$

**Figure 2:**
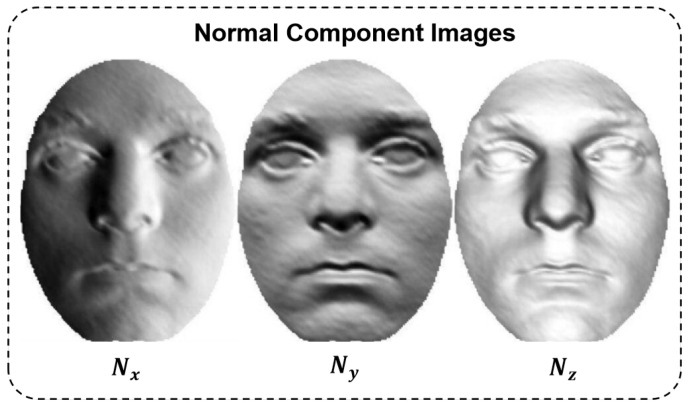
Illustration of facial normal estimation: the original range image and its normal component images ($N_{x}$, $N_{y}$, $N_{z}$).

These normal component images encode fine-grained local geometry and preserve topological cues from the original 3D surface. As shown in Figure 2, key facial regions such as the eyes, nose, and mouth exhibit high variation in surface orientation, making normal component images particularly effective for identity recognition. This results in three specialized feature extractors, each attuned to a specific directional surface encoding.

During fine-tuning, we explore two types of classification losses: the standard cross-entropy loss and the large margin cosine loss [12], which is widely used in face recognition tasks to improve inter-class separability and intra-class compactness in the angular space. The large margin cosine loss (LMCL) introduces an angular margin to enhance inter-class separability and intra-class compactness by operating in the normalized feature space. The loss is defined as

(8) $$L_{\mathrm{LMCL}}=-\log\left(\frac{e^{s(\cos\left(\theta_{y}\right)-m)}}{e^{s(\cos\left(\theta_{y}\right)-m)}+\sum_{j\neq y}e^{s\cos(\theta_{j})}}\right),$$

where $\theta_{j}$ denotes the angle between the normalized feature vector f and the normalized weight vector $W_{j}$ of class *j*; $\theta_{y}$ corresponds to the ground-truth class *y*. The scalar *s* is a fixed scaling factor that controls the magnitude of the logits, and *m* is the additive angular margin used to explicitly enforce a larger separation between classes in angular space. By explicitly optimizing the angular decision boundaries, the large margin cosine loss produces more discriminative features. In practice, we found that fine-tuning with the large margin cosine loss leads to improved recognition accuracy compared to standard cross-entropy.

### 2.4. Deep 3D Face Identification

During the test, each 3D face of the probe and the gallery is transformed similarly into its corresponding normal component images and passed through the fine-tuned deep face recognition model to extract its deep features. To obtain discriminative features from the normal component images along each axis, we employ three independent deep face recognition models fine-tuned on $N_{x}$, $N_{y}$, and $N_{z}$, respectively:

(9) $$DNP_{x}=f_{x}\left(N_{x}\right),$$

(10) $$DNP_{y}=f_{y}\left(N_{y}\right),$$

(11) $$DNP_{z}=f_{z}\left(N_{z}\right),$$

where $f_{x},f_{y},f_{z}$ denote the axis-specific deep face recognition models. Each model extracts a deep normal pattern (DNP) corresponding to the *x*-, *y*-, and *z*-axis normal components. Face identification is then performed by computing the Euclidean distance between the DNPs of the probe and gallery samples along each axis.

(12) $$d_{x}={∥DNP_{x}^{\left(p\right)}-DNP_{x}^{\left(g\right)}∥}_{2},$$

(13) $$d_{y}={∥DNP_{y}^{\left(p\right)}-DNP_{y}^{\left(g\right)}∥}_{2},$$

(14) $$d_{z}={∥DNP_{z}^{\left(p\right)}-DNP_{z}^{\left(g\right)}∥}_{2}$$

Finally, the three scores are fused at the decision level using simple averaging to obtain the final similarity score:

(15) $$d=\frac{1}{3}\left(d_{x}+d_{y}+d_{z}\right)$$

The identity corresponding to the lowest fused distance is selected as the final prediction. This score-level fusion strategy integrates complementary geometric cues along orthogonal surface directions and has proven effective for robust 3D face identification.

## 3. Experiments

This section presents a comprehensive evaluation of our proposed approach. We first introduce the training and test datasets, as well as evaluation protocols. Then, we assess the effectiveness of the reconstructed 3D faces in face identification tasks. Finally, we compare our method against several state-of-the-art approaches on public 3D face datasets.

### 3.1. Datasets

The VGGFace2 database [15] contains 3.31 million face images from 9131 subjects, collected from Google. It features a wide variety of ages, poses, lighting conditions, races, and occupations. In addition to identity labels, it includes facial bounding boxes, five facial landmarks, as well as estimated age and pose annotations. Standard data augmentation techniques (e.g., horizontal flipping, and random cropping) are applied.

The BU-3DFE database [16] consists of 2500 3D facial scans from 100 subjects. Each subject displays six prototypical expressions (happiness, disgust, fear, anger, surprise, and sadness) at four intensity levels, in addition to a neutral expression. The neutral scans are used to form the gallery, while the remaining 2400 scans serve as probes. BU-3DFE is widely recognized as a challenging benchmark due to its expression diversity.

The FRGC v2 database [17] comprises 4007 textured 3D face scans from 466 subjects, including 1642 samples with varying facial expressions, all captured under controlled lighting. Each face scan has a resolution of 640×480. The first scan of each subject is used for the gallery, and the remaining scans serve as probes.

The Bosphorus database [18] contains 4666 3D scans from 105 individuals (60 male, 45 female), covering variations in head pose, expression, and occlusion. For this work, we adopt a subset of 2902 scans containing only expression variations. The first neutral scan of each subject is used as the gallery, while the remaining 2797 scans are used as probes.

The BU-4DFE database [19] contains 101 subjects (58 females, 43 males) with expressions of anger, happiness, fear, disgust, sadness, surprise, and neutral. Each expression is represented by a 3D sequence with a length of around 100 frames. Each sequence is supposed to begin with neutral expression, then change to the labeled expression with strong expression intensity, and finally return to neutral expression. To compare fairly, we adopt the strategy that retains five frames equally spaced apart for each sequence. Among retained frames, the first frame per identity is selected to form the gallery (101 scans) and others as the probes (2929 scans).

In our experiments, the VGGFace2 dataset is utilized for training. We first pre-train the Sphere20 architecture on the original 2D images from the VGGFace2 dataset. Subsequently, we reconstruct 3D face scans from these 2D images and convert the scans into their corresponding normal component images. These normal component images are then employed to fine-tune the pre-trained Sphere20 model. Importantly, no real 3D face data are used during training. Instead, the model is trained exclusively on 2D images and their reconstructed 3D face data.

### 3.2. Effectiveness of Reconstructed 3D Face Scans

To evaluate the effectiveness of the proposed 2D-aided deep 3D face identification framework, we assess the quality of reconstructed 3D faces by examining their contribution to identification performance across three widely used 3D face datasets. During pre-training, RGB face images of size 112×112 from the VGGFace2 dataset are fed into the Sphere20 network, which outputs 9131-dimensional vectors representing class probabilities. In the fine-tuning phase, the fully connected and softmax layers are re-initialized and optimized. Cross-entropy loss is used consistently in both the pre-training and fine-tuning stages. It is worth noting that the baseline results are obtained by the pre-trained Sphere20 model without any adaptation to 3D geometry. In contrast, our proposed method fine-tunes this pre-trained model using the reconstructed 3D normal component images, which encode surface orientation along three axes and serve as a bridge between 2D convolutional neural network and 3D geometry.

The rank-1 accuracies on the BU-3DFE dataset are presented in Table 1. Incorporating normal component images ($N_{x}$, $N_{y}$, $N_{z}$) significantly improves performance. Specifically, the accuracy improves from 66.1% to 92.2% along the *x*-axis, from 71.0% to 94.2% along the *y*-axis, and from 75.5% to 96.5% along the *z*-axis. Furthermore, late fusion of all three directional features boosts the accuracy to 98.0%, demonstrating the complementary nature of the three components. Consistent performance gains are observed on the FRGC v2 dataset (Table 2), where the accuracy improves from 73.8% to 86.7% ($N_{x}$), 78.6% to 84.1% ($N_{y}$), and 77.4% to 87.1% ($N_{z}$), with fusion yielding 90.2%. Similarly, on the Bosphorus dataset (Table 3), our method achieves substantial improvements over the baseline: from 70.0% to 94.2% ($N_{x}$), 80.8% to 96.5% ($N_{y}$), and 78.4% to 96.5% ($N_{z}$). The late fusion strategy again provides the best performance, achieving a rank-1 accuracy of 98.0%. These results consistently demonstrate that the proposed framework, which leverages 2D images and their reconstructed 3D representations, can effectively enhance 3D face identification without requiring real 3D training data.

To evaluate the adaptability of our 2D-to-3D augmentation pipeline to different 3D face reconstruction methods, we replaced ExpNet with a recent diffusion-based method, i.e., TEx-Face [20]. We then retrained the recognition model on the synthetic 3D face data of TEx-Face and evaluated it on the same benchmarks. As shown in Table 4, Table 5, Table 6 and Table 7, TEx-Face consistently improves recognition accuracy over ExpNet. These results indicate that our framework is not only compatible with different reconstruction pipelines but also benefits from higher-fidelity geometric inputs. This highlights the flexibility and extensibility of the proposed approach, which can seamlessly integrate stronger 3D reconstruction models to further enhance 3D face recognition performances.

### 3.3. Comparison with State-of-the-Art 3D Face Recognition Methods

To further enhance identification performance, we apply the large margin cosine loss (LMCL) [31] during the fine-tuning stage. Table 4, Table 5 and Table 6 present the rank-1 accuracies of our method in comparison with state-of-the-art 3D face recognition approaches on the BU-3DFE, FRGC v2, and Bosphorus datasets, respectively.

Our method achieves rank-1 accuracies of 99.2%, 98.4%, 99.3%, and 96.5% on the four datasets, consistently outperforming most prior methods and achieving comparable performance to state-of-the-art approaches across all benchmarks. Notably, our model maintains consistently high accuracy without relying on direct supervision from real 3D data, demonstrating strong generalizability and robustness. This demonstrates the effectiveness of our 2D-aided framework in leveraging normal component images to boost 3D face identification.

Although our method does not achieve the absolute highest accuracy on all benchmarks, it is important to note that the entire training pipeline relies solely on 2D face images and their 3D reconstructions, without access to any real 3D face scans. In contrast, many competing approaches utilize real 3D scans, private datasets, or dataset-specific augmentation strategies. For instance, Cai et al. [24] and Yu et al. [9] adopt private 3D face scans as training data, and apply augmentations to real 3D scans. Zhu et al. [29] address the challenge of occluded 3D face recognition by extracting multiple features from point clouds and depth maps, and applying a threshold-based fusion strategy to enhance robustness under occlusions. These techniques, although effective, depend on carefully curated 3D face scans. Compared to methods that rely on real 3D face scans, our framework achieves competitive recognition performance in a scalable and resource-efficient way, despite being trained solely on reconstructed 3D face data.

### 3.4. Evaluation on a Large-Scale 3D Face Dataset

While benchmark datasets such as BU-3DFE and Bosphorus offer valuable evaluation settings, they are relatively limited in the number of subjects and 3D scans. To better assess the scalability and generalizability of our approach, we construct a large-scale test set by merging the BU-3DFE, FRGC v2, and Bosphorus datasets. The resulting combined dataset contains 671 unique subjects in the gallery and a total of 8738 3D facial scans in the probe set. As reported in Table 8, our method achieves a high rank-1 accuracy of 98.2% on this challenging large-scale setting. This result highlights the robustness, scalability, and generalization capability of our 2D-aided 3D face recognition framework, even in the presence of significant subject diversity and data heterogeneity across datasets.

## 4. Discussion

In this study, we have demonstrated the effectiveness of a 2D-aided 3D face identification framework based on reconstructed 3D face scans. The experimental results across multiple public 3D face datasets show that even without using real 3D scans in training, our approach can achieve high identification accuracy, which highlights the potential of leveraging large-scale 2D face datasets for 3D face recognition tasks.

One of the key advantages of our method lies in its scalability. By avoiding the need for expensive and hard-to-acquire 3D facial data during training, the framework can be easily adapted and deployed in real-world scenarios where only 2D face images are available. In addition, we observe that our method maintains high and stable performance even on a merged large-scale test set, suggesting good generalization ability.

However, our approach also has certain limitations. The performance is dependent on the quality of the reconstructed 3D faces, which may vary depending on the reconstruction model used. In this work, only ExpNet was employed for 3D reconstruction. Moreover, the current framework treats reconstruction and recognition as two separate stages, which may not be optimal for end-to-end learning.

In future work, we plan to explore joint training strategies where the 3D reconstruction and recognition modules are optimized together. We also intend to evaluate more advanced or task-specific 3D reconstruction methods and investigate whether recognition-driven reconstruction can further improve identification accuracy. Finally, we will consider extending our framework to more diverse face recognition scenarios, such as cross-pose or occluded face recognition.

## 5. Model Complexity

To assess the practical efficiency of our pipeline, we measured both inference time and memory usage on a single NVIDIA GeForce RTX 3090 GPU. ExpNet uses a ResNet-101 backbone to regress 3DMM parameters from a single RGB input. Over 1000 runs, the average inference time is 0.065 s per image, and peak GPU memory consumption is 278 MB. Our recognition stage processes normal component images through three independent Sphere20 branches. Each branch requires 0.020 s for a forward pass. When executed in parallel, the combined inference time is 0.025 s. The total memory usage for all three branches is 375 MB. Because 3D reconstruction is only performed during training, the deployed system’s runtime cost is limited to the recognition module.

## 6. Conclusions

This work presents a preliminary exploration of 2D-aided 3D face recognition, demonstrating that high-quality 3D reconstructions from large-scale 2D face images can effectively support deep 3D recognition. Without relying on any real 3D scans, our pipeline achieves competitive performance across multiple benchmarks, underscoring its practicality and scalability for scenarios where large-scale 3D data acquisition is costly or impractical.

While our main framework employs ExpNet as the reconstruction module, additional experiments using a more advanced method, TEx-Face, demonstrate that improvements in reconstruction fidelity consistently lead to enhanced 3D face recognition accuracy. These findings underscore the potential of future advancements in geometric modeling to further strengthen the performance of 3D face recognition.

We also acknowledge certain limitations of using synthetic 3D face data. Although our approach proves effective, it may not fully capture the fine-grained geometric variations and surface-level imperfections present in real 3D scans, which can affect generalization to unconstrained real-world data. In future work, we plan to explore recognition-aware reconstruction, end-to-end optimization of the full pipeline, and hybrid training strategies that incorporate a small amount of real 3D data to bridge this domain gap.

Our findings suggest that 2D-to-3D synthetic training is a viable and efficient paradigm for deep 3D face recognition, and we believe this direction holds substantial potential for further research and practical deployment. Furthermore, our 2D-aided framework is naturally extensible to multimodal settings, such as combining 2D RGB, depth, or infrared modalities. Recent multimodal methods such as GD-YOLO [32] illustrate complementary strategies that can be integrated with our framework in future work.

## Figures and Tables

**Table 1:** The rank-1 accuracy improvements in 3D face identification on the BU-3DFE database.

	Baseline	Ours
$N_{x}$	66.1%	92.2%
$N_{y}$	71.0%	94.2%
$N_{z}$	75.5%	96.5%
Late Fusion	80.3%	98.0%

**Table 2:** The rank-1 accuracy improvements in 3D face identification on the FRGC v2 database.

	Baseline	Ours
$N_{x}$	73.8%	86.7%
$N_{y}$	78.6%	84.1%
$N_{z}$	77.4%	87.1%
Late Fusion	81.3%	90.2%

**Table 3:** The rank-1 accuracy improvements in 3D face identification on the Bosphorus database.

	Baseline	Ours
$N_{x}$	70.0%	94.2%
$N_{y}$	80.8%	96.5%
$N_{z}$	78.4%	96.5%
Late Fusion	84.6%	98.0%

**Table 4:** The comparison of rank-1 accuracy on the BU-3DFE database. Bold values indicate the best results.

Method	Rank-1 Score
Li et al. [21]	92.2%
Lei et al. [22]	94.0%
Li et al. [23]	96.1%
Kim et al. [7]	95.0%
Gilani et al. [10]	98.6%
Cai et al. [24]	99.9%
Yu et al. [9]	**100.0%**
Ours (ExpNet)	98.8%
Ours (TEx-Face)	99.2%

**Table 5:** The comparison of rank-1 accuracy on the FRGC v2 database. Bold values indicate the best results.

Method	Rank-1 Accuracy
Li et al. [21]	96.3%
Lei et al. [22]	96.3%
Li et al. [23]	98.0%
Gilani et al. [10]	97.1%
Wang et al. [25]	96.0%
Ours (ExpNet)	97.6%
Ours (TEx-Face)	**98.4%**

**Table 6:** The comparison of rank-1 accuracy on the Bosphorus database. Bold values indicate the best results.

Method	Rank-1 Accuracy
Li et al. [21]	95.4%
Li et al. [23]	97.6%
Kim et al. [7]	99.2%
Gilani et al. [10]	96.2%
Yu et al. [8]	99.3%
Cai et al. [24]	**99.8%**
Yu et al. [9]	**99.8%**
Wang et al. [25]	92.0%
Zhao et al. [26]	98.9%
Yang et al. [27]	97.9%
Wang et al. [28]	98.9%
Zhu et al. [29]	99.5%
Ours (ExpNet)	98.4%
Ours (TEx-Face)	99.3%

**Table 7:** The comparison of rank-1 accuracy on the BU-4DFE database. Bold values indicate the best results.

Method	Rank-1 Accuracy
Gilani et al. [30]	96.0%
Gilani et al. [10]	95.5%
Yu et al. [9]	**98.0%**
Ours (ExpNet)	96.1%
Ours (TEx-Face)	96.5%

**Table 8:** The rank-1 accuracy of our method on the large-scale database.

Method	Rank-1 Accuracy
Ours	98.2%

## Data Availability

The data presented in this study are available online.
